# Geoditin A Induces Oxidative Stress and Apoptosis on Human Colon HT29 Cells

**DOI:** 10.3390/md8010080

**Published:** 2010-01-19

**Authors:** Florence W. K. Cheung, Chunman Li, Chun-Tao Che, Bonnie P. L. Liu, Lijun Wang, Wing-Keung Liu

**Affiliations:** 1 School of Chinese Medicine, the Chinese University of Hong Kong, Shatin, N.T., Hong Kong, China; E-Mails: Florence_cwk@hotmail.com (F.W.K.C.); lichunmn2000@hotmail.com (C.L.); chect@cuhk.edu.hk (C-T.C.); 2 School of Biomedical Sciences, Faculty of Medicine, the Chinese University of Hong Kong, Shatin, New Territories, Hong Kong, China; E-Mails: bonniepliu@gmail.com (B.P.L.L.); s063580@cuhk.edu.hk (L.W.)

**Keywords:** geoditin A, apoptosis, transferrin receptor, oxidative stress

## Abstract

Geoditin A, an isomalabaricane triterpene isolated from the marine sponge *Geodia japonica*, has been demonstrated to dissipate mitochondrial membrane potential, activate caspase 3, decrease cytoplasmic proliferating cell nuclear antigen (PCNA), and induce apoptosis of leukemia cells, but the underlying mechanism remains unclear [1]. In this study, we found fragmentation of Golgi structure, suppression of transferrin receptor expression, production of oxidants, and DNA fragmentation in human colon cancer HT29 cells after treatment with geoditin A for 24 h. This apoptosis was not abrogated by chelation of intracellular iron with salicylaldehyde isonicotinoyl hydrazone (SIH), but suppressed by *N*-acetylcysteine (NAC), a thiol antioxidant and GSH precursor, indicating that the cytotoxic effect of geoditin A is likely mediated by a NAC-inhibitable oxidative stress. Our results provide a better understanding of the apoptotic properties and chemotherapeutical potential of this marine triterpene.

## 1. Introduction

Colorectal cancer has traditionally been one of the four leading death cancers worldwide, but the incidence was relatively low in Asian populations. However, the prevalence of colorectal neoplasm has increased by 2- to 4-fold in some developed Asian countries, including China, Japan, South Korea, and Singapore in the past few decades [2], due to changes in dietary habits and lifestyle as well as certain genetic factors of Asian populations [2]. In Hong Kong there were 3,918 new bowel cancer cases and 1,628 deaths registered in 2006 [3], making colorectal cancer the second most common cancer and it is expected to surpass lung cancer to become the most common cancer within the next five years [3]. Surgical resection and chemotherapy are two common treatment strategies for early stage colon cancer, but recurrence from chemo-resistance or adverse side effects of conventional chemotherapeutic agents, such as cytotoxicity of 5-fluorouracil, not only hamper the therapeutic regimen but also affect the quality of life of the patients, therefore novel bioactive agents from natural sources are urgently needed for effective chemotherapy [4].

The search for therapeutic agents or bioactive natural compounds from territorial plants and microbes had a long tradition up to the initiation of the symposium “Drugs from the Sea” in 1968 [5], and since then over 16,000 different natural products have been isolated from marine organisms in the past decades [6]. These marine compounds are diverse in chemical structures and biological activities, and ziconotide (Prialt^®^), a peptide from a tropical cone snail for the treatment of pain, was approved in the United States in 2004 [7], and trabectedin (Yonledis^®^) from the Caribbean tunicate *Ecteinascidia turbinata* was approved in the European Union in 2007 for treatment of ovarian cancer [8]. Isomalabaricanes, tricyclic terpenoids isolated from many genera of marine sponges, have received special pharmaceutical attention because of their inhibitory activities towards the cyclin-dependent kinases and controlling tumor cell cycle proliferation, implicating the potential application of marine compounds for chemotherapy [8,9].

Iron is an essential element to the body acting as a cofactor of heme proteins for a wide variety of cellular processes such as metabolism, respiration, and DNA synthesis. It forms a complex with transferrin before they are delivered into the cells via interactions with surface transferrin receptor (TfnR)[10]. TfnR is constitutively expressed in cancer cells, up to 4- to 5-fold higher than nonneoplastic cells because of the need for iron as a cofactor of DNA synthesis of rapidly dividing cells. Once the iron is released from the Tfn, the Tfn-TfnR complex is recycled either directly or indirectly through the Golgi complex back to the cell surface [10]. Abnormal expression of Tfn/TfnR results in an unbalanced iron homeostasis that is associated with oxidative stress and programmed cell death [10] which has been well documented in HT29 cells treated with flavone [11], or tangutorine [12]. In this study an apoptosis associated with a decrease of transferrin receptors and oxidative stress were induced by geoditin A in colon cancer HT29 cells, and this apoptosis was diminished by pre-treatment with oxidant scavenger, *N*-acetylcysteine, implicating the apoptosis inducing activity of geoditin A is mediated through oxidative stress.

## 2. Results and Discussion

**Figure 1:**
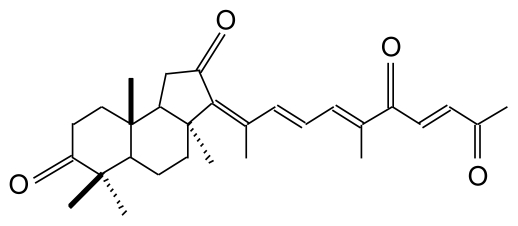
Chemical structure of geoditin A.

Geoditin A (Figure 1) is an isomalabaricane triterpene with three sets of α,β-unsaturated ketone structures, first isolated from *Stelletta tenuis* [13], but also extracted from a marine sponge, *Geodia japonica*, collected from South China Sea [14]. There is increasing evidence that isomalabaricane is a rare class of triterpenoids with potent tumor inhibitory activities [15,16], the values of IC_50_ for cancer cell lines ranging from 0.1–20 μg/mL, which has brought attention to its potential for chemotherapeutic development [17].

Our previous study has also demonstrated a potent cytotoxicity of geoditin A against human leukemia HL60 cells (IC_50_ = 3 μg/mL), in which a dissipation of mitochondrial membrane potential, activation of caspase 3, and decrease of cytoplasmic proliferating cell nuclear antigen (PCNA) were revealed by fluorescence microscopy [1].

**Figure 2:**
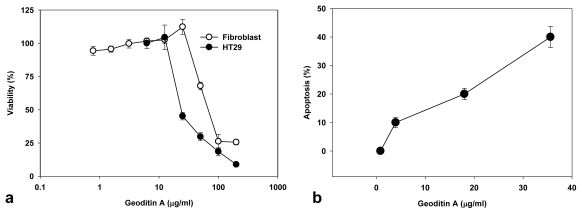
Cytotoxicity and apoptotic activity of geoditin A on HT29 cells and human dermal fibroblasts. Cells were incubated in 96-well plates with serial concentrations of geoditin A for 24 h, and its cytotoxicity was assessed by MTT bioassay (a). Results were presented as mean and standard deviation of triplicates. Treated cells were fixed in cold 70% alcohol and stained with propidium iodide before the subG1 population (apoptosis) was measured by flow cytometry (b).

The treated cells also manifested nuclear fragmentation typical for apoptosis, but autophagic change was not prominent although compounds with α,β-unsaturated ketone framework may induce autophagy [18], and the underlying mechanisms mediating these changes remain unclear. Geoditin A showed a dose-dependent cytotoxicity in human colon HT29 cells as determined by the MTT bioassay, and the value of IC_50_ is 20 μg/mL, which is at least 2 folds higher than that (IC_50_ = 60 μg/mL) for human dermal fibroblasts (Figure 2a), indicating its specificity for cancer cells. Increase of apoptosis from 10% to 40% was measured in HT29 cells treated for 24h with geoditin A from 5 to 40 μg/mL by flow cytometry (Figure 2b).

**Figure 3:**
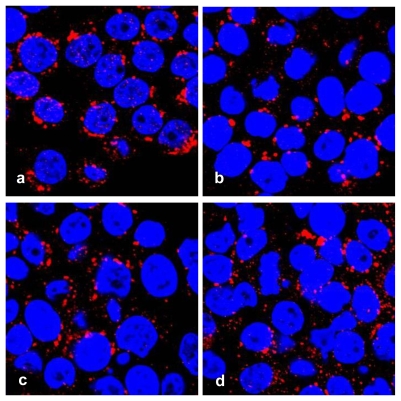
Golgi fragmentation in apoptotic HT29 cells. Cells were treated with serial concentrations of geoditin A for 24 h, fixed in 3% paraformaldehyde (PFA) and incubated with antibody against Golgi marker protein, golgin-97 and visualized by secondary antibody IgG conjugated to Alexa-647. Nuclei were counterstained with DAPI. Typical Golgi stack is shown at the juxtanuclear region of the untreated HT29 cells (a), while Golgi fragmentation becomes prominent in geoditin A-treated in a dose-dependent manner (b, c, and d for 10, 20 and 40 μg/mL, respectively).

Features for apoptosis, including nuclear fragmentation by DAPI staining, and Golgi fragmentation by immunofluorescence with antibody against golgin-97, a marker protein for the Golgi complex [19] were visualized in treated HT29 cells with a confocal microscope (Figure 3).

**Figure 4:**
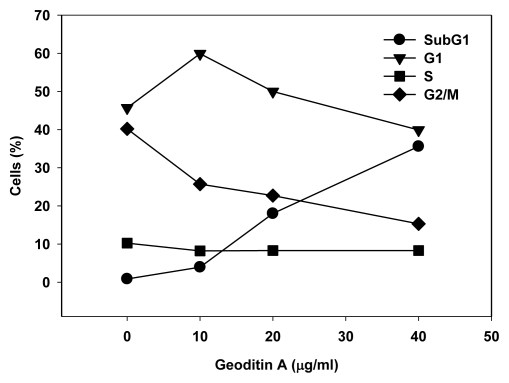
Change of DNA patterns in geoditin A-treated HT29 cells. Treated cells were fixed in cold 70% alcohol and stained with propidium iodide before their DNA patterns were analyzed by flow cytometry. SubG1 cell population increased in the expenses of G2/M populations.

The Golgi apparatus is not only a vital organelle for protein synthesis and post-translational modification, but can also regulate early apoptotic events [20]. The Golgi proteins, e.g., golgin-160 and p115, are disassembled into fragments by proteolytic action of upstream caspases and followed by the nuclear translocation of the C-terminal fragment of p115 for an induction of apoptosis. It will be interesting to further study if a similar cell death pathway is shared by geoditin A. Flow cytometric analysis further confirmed an elevated subG1 population from 20% to 40% after treatment of HT29 cells for 20 μg/mL and 40 μg/mL geoditin A, respectively (Figure 2b). The increase of subG1 population mainly with the expense of G2/M cells implicated a decrease of cell growth by geoditin A treatment (Figure 4).

Iron is an essential element for normal cell growth and metabolism, but a higher level of iron is required by malignant cells for their proliferation and progression. The increase of cellular iron uptake is associated with an increased expression of iron import proteins, such as Tfn and TfnR [21]. Iron in the serum is coupled to Tfn before endocytosis into the cell via interaction with its ligand receptors on the plasma membrane, and iron is then released from the Tfn-TfnR complex in the endosomes before both Tfn and TfnR are returned to the plasma membrane through recycling endosomes [11].

**Figure 5:**
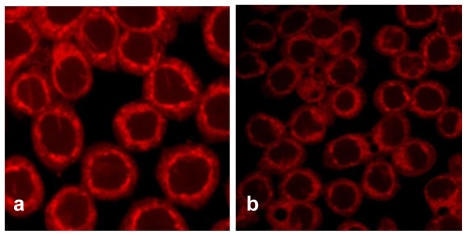

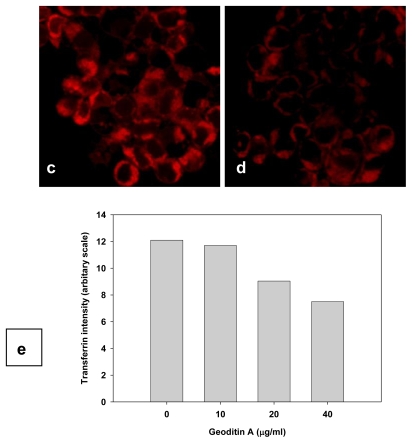
Suppression of Tfn endocytosis by geoditin A. HT29 cells were treated with geoditin A for 24 h, washed with warm plain DMEM medium with 1% BSA, incubated with fresh DMEM containing 50 μg/mL Alexa-594 Tfn for 60 min, before the cellular uptake of Tfn was observed under a fluorescence microscope. Representative images were captured using FV1600 Olympus system. Tfn was located in endosomes of untreated cells (a) but decreased in a dose-dependent manner after treatment with geoditin A for 24 h (b, c, d for 10, 20 and 40 μg/mL, respectively). About 30% decrease of Alexa-594 Tfn was measured in HT29 cells treated with geoditin A for 40 μg/mL by flow cytometry (e).

**Figure 6:**
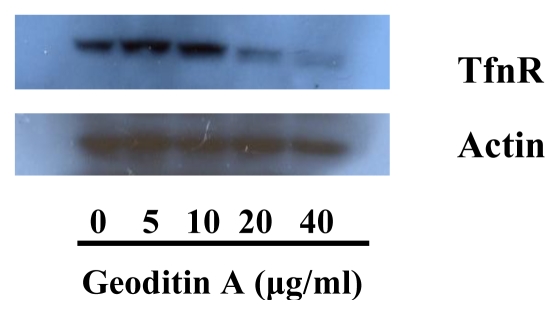
Down-regulation of transferrin receptor (TfnR) expression in geoditin A-treated HT29 cells. Treated HT29 cells were lysed and total protein lysate was subjected to separation on a 10% SDS-PAGE gel, transferred onto a PVDF membrane and probed with antibodies against TfnR and β-actin.

TfnR is up-regulated in cancer cells for enhancing iron uptake, and iron depletion by iron chelators or decreased expression of Tfn and TfnR have been proven to be effective in the treatment of cancer [10,21]. Tfn endocytosed into HT29 cells and accumulated in the early/late and recycling endosomes in the cytoplasm (Figure 5a), but the level of endocytic Tfn-fluorescence decreased in a dose-dependent manner in geoditin A-treated cells (Figures 5b–d), and a decrease of >30% labeled transferrin in these cells was measured by flow cytometric analysis (Figure 5e). Immunoblotting analysis also showed a significant down-regulation of iron carriers, TfnR, in HT29 cells after treatment with geoditin A for 24 h, particularly at ≥20 μg/mL (Figure 6).

Reactive oxygen species (ROS) are by-products of aerobic metabolism which play important role in intracellular signaling cascades. Mitochondria and NADPH oxidase are two major sources for intracellular ROS whose overproduction can activate stress pathway that ultimately leads to cell apoptosis [22]. Intracellular iron is also a pro-oxidant catalyst which induces oxidative stress via Fenton/Haber-Weiss reaction [23], DNA strand breakage and cell death [24]. Iron chelators, e.g., salicylaldehyde isonicotinoyl hydrazone (SIH), binds intracellular labile iron, reduces iron-catalyzed hydroxyl radical generation, and thus protects cells from apoptosis [25]. In order to delineate if apoptotic changes of HT29 cells in this study was mediated by oxidative stress or iron overload, cells were intervened with antioxidants or iron chelators before apoptosis was induced by 20 μg/mL geoditin A for 24 h.

**Figure 7:**
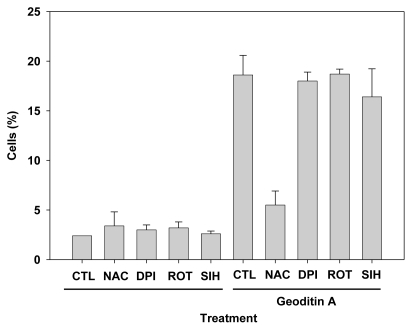
Suppression of HT29 apoptosis by oxidant scavenger NAC. HT29 cells were pre-incubated with oxidant inhibitors, NAC (*N-*acetylcysteine, 10 mM), diphenyleneiodonium chloride (DPI, 1 μM) and rotenone (ROT, 5 μM), or iron chelator SIH (5 μM) for 2 h and further co-cultured for 24 h with 20 μg/mL geoditin A before their apoptosis cells were measured by flow cytometry. Treatment of cells with oxidative inhibitors or SIH alone did not show significant differences with untreated control. Treatment of geoditin A-treated cells with NAC significantly reduced apoptosis from 19% to only 5.5%, whereas DPI (18%), Rot (18.7%), and SIH (16.4%) pre-treatment could not protect cells from apoptosis (19% for cells treated with geoditin A). Cells without geoditin A treatment served as controls (CTL). Results were presented as mean and standard deviation of triplicates.

Results of flow cytometry with these cells show that 20% of HT29 cells had undergone apoptosis which could not be rescued by oxidant inhibitors, e.g., diphenyleneiodonium chloride (DPI) and rotenone for NADPH oxidase and electron transport in complex I of mitochondria, respectively (data not shown), or iron chelation with 5 μM SIH, but pre-treatment of 10 mM *N*-acetylcysteine (NAC) could reduce apoptosis by more than 60% (Figure 7), implicating a major role of oxidative stress in an induction of apoptotic changes in HT29 cells. Taken our results together, we have demonstrated that geoditin A induced oxidative stress in HT29 cells and elicited its cytotoxicity through a *N*-acetylcysteine inhibitable apoptosis.

## 3. Experimental Section

### 3.1. Test compounds

Geoditin A is an isomalabaricane triterpene isolated from a marine sponge, *Geodia japonica* [14], and it has a molecular weight of 450 and a molecular formula of C_20_H_26_O_4_ as determined by mass spectrometry and elemental analysis [14]. The compound was dissolved in DMSO to make a stock solution at a concentration of 40 mg/mL which was then diluted to appropriate concentrations with culture medium before each experiment. The final concentration of DMSO did not exceed 0.5% in any experiment. Iron chelator, salicylaldehyde isonicotinoyl hydrazone, was a kind gift of Dr. P Ponka of Jewish General Hospital, Montreal, Canada.

### 3.2. Cell cultures

Human colon cancer HT29 (HTB-38, ATCC) cells and human dermal fibroblasts were routinely maintained in RPMI-1640 and DMEM, respectively, supplemented with 10% fetal bovine serum (FBS), 100 μg/mL streptomycin and 100 IU/mL penicillin at 37°C in a humidified atmosphere of 5% CO_2_.

### 3.3. Cell proliferation assay

HT29 cells and dermal fibroblasts (2 × 10^4^ cells/0.1 mL/well) were treated with a serial dilution of geoditin A in 96-well culture plates (Costar, USA) or 8-chamber culture slides (Nunc 177402, USA) for 48 h. During the last 4 h, cells were reacted with MTT (3-[4,5-dimethylthiazol-2-yl]-2,5 diphenyl tetrazolium bromide) at 37 °C for colorimetric MTT-based cytotoxicity assay. The reaction product, formazan, was extracted with DMSO and the absorbance was read at 540 nm [12]. Data represent the mean values and standard deviations of triplicate assays in at least one experiment.

### 3.4. Fluorescence staining for morphological observation

HT29 cells were treated with a serial dilution of geoditin A in 8-chamber slides for 24 h, washed briefly with phosphate-buffered saline (PBS) before they were fixed with buffered formalin, stained with antibody against golgin-97 (A21270, Invitrogen, USA), a marker for Golgi complex, followed by secondary antibody IgG conjugated to Alexa-647 in TBS-T buffer, and the chromatin was counter-stained with DAPI before the slides were mounted with anti-fade for microscopy on a confocal microscope (Axioskop, Zeiss, Japan) with a 450–490 nm excitation block filter and a 520 nm barrier filter [12].

### 3.5. Flow cytometric cell cycle analysis

Cells were treated with geoditin A for 24 h and a cell suspension of 100,000 cells was fixed with 70% alcohol for 15 min at 4 °C, treated with RNase A and stained with 1.0 μg/mL propidium iodide (PI, Boehringer Mannheim, Germany). The red fluorescence of DNA-bound PI in individual cells was measured at 488 nm with a Beckman Coulter Altra flow cytometer and the results were analyzed using Expo32 software (Beckman Coulter, USA)[12]. Cells pre-incubated with diphenyleneiodonium chloride (DPI, 1 μM), rotenone (ROT, 5 μM), NAC (10 mM), or SIH (5 μM) for 2 h before co-cultured with 20 μg/mL geoditin A for 24 h were subjected to apoptosis analysis by flow cytometry to elucidate the source of ROS production [12], and the effect of intracellular iron.

### 3.6. Immunoblotting analysis

HT29 cells at 24 h after 2h-drug exposure were washed with PBS twice, and the total protein lysates were obtained in lysis buffer (50 mM Tris-Cl, 150 mM NaCl, 0.2% Triton X-100, 10 μg/mL aprotinin and 0.5 mM PMSF), and centrifuged at 10000 rpm at 4 °C for 10 min. Lysates were normalized for protein content using the protein assay reagent (500-0006, Bio-Rad Laboratories, USA). Equal amounts of denatured proteins were loaded and separated on a 10% SDS polyacrylamide gel, and were then transferred to a polyvinylene difluoride (PVDF) membrane. After blocking with 2% gelatin, the membrane was stained with specific primary antibodies against transferrin receptor (13–6800 Zymed Laboratories, South San Francisco CA, USA) and actin, respectively, followed by secondary antibody IgG conjugated to horseradish peroxidase in TBS-T buffer. The signals were detected using the ECL^™^Plus Western Blotting Analysis System (Amersham Pharmacia Biotech, Piscataway, NJ, USA**),** followed by short exposures to Lumi-film Chemiluminescence Detection Film (Roche Diagnostics Corporation, Indianapolis, IN, USA). Band intensities were quantified by the software PD Quest (BioRad Laboratories, Hercules, CA, USA) and normalized by β-actin [12].

### 3.7. Internalization of transferrin by Geoditin A-treated HT29 cells

HT29 cells were pre-treated with serial concentrations of geoditin A for 24 h, depleted endogenous transferrin by incubating in plain DMEM containing 1% BSA for 1 h before transferrin uptake assay was performed for 1 h in DMEM containing 50 μg/mL Alexa-594 transferrin. The cells were then washed two times with ice-cold PBS, fixed with 2% PFA for 20 min and then subjected to confocal fluorescence microscopy. Another portion of cells (20,000/group) treated the same way were subjected to flow cytometric measurement of transferrin uptake.
